# Development of a patient-centred intervention to improve knowledge and understanding of antibiotic therapy in secondary care

**DOI:** 10.1186/s13756-018-0333-1

**Published:** 2018-03-20

**Authors:** Timothy M. Rawson, Luke S. P. Moore, Enrique Castro-Sanchez, Esmita Charani, Bernard Hernandez, Vivian Alividza, Fran Husson, Christofer Toumazou, Raheelah Ahmad, Pantelis Georgiou, Alison H. Holmes

**Affiliations:** 1 National Institute for Health Research Health Protection Research Unit in Healthcare Associated Infections and Antimicrobial Resistance, Imperial College London, Hammersmith Campus, Du Cane Road, London, W12 0NN UK; 2 Imperial College Healthcare NHS Trust, Du Cane Road, London, W12 0HS UK; 3 Chelsea & Westminster Hospital, 369 Fulham Rd, Chelsea, London, SW10 9NH UK; 4 Centre for Bio-Inspired Technology, Imperial College London, South Kensington Campus, Exhibition Road, London, SW7 2AZ UK

**Keywords:** Patient & Public Involvement, Co-design, Antimicrobial prescribing, Shared-decision making

## Abstract

**Background:**

We developed a personalised antimicrobial information module co-designed with patients. This study aimed to evaluate the potential impact of this patient-centred intervention on short-term knowledge and understanding of antimicrobial therapy in secondary care.

**Methods:**

Thirty previous patients who had received antibiotics in hospital within 12 months were recruited to co-design an intervention to promote patient engagement with infection management. Two workshops, containing five focus-groups were held. These were audio-recorded. Data were analysed using a thematic framework developed deductively based on previous work. Line-by-line coding was performed with new themes added to the framework by two researchers. This was used to inform the development of a patient information module, embedded within an electronic decision support tool (CDSS).

The intervention was piloted over a four-week period at Imperial College Healthcare NHS Trust on 30 in-patients. Pre- and post-intervention questionnaires were developed and implemented to assess short term changes in patient knowledge and understanding and provide feedback on the intervention. Data were analysed using SPSS and NVIVO software.

**Results:**

Within the workshops, there was consistency in identified themes. The participants agreed upon and co-designed a personalised PDF document that could be integrated into an electronic CDSS to be used by healthcare professionals at the point-of-care. Their aim for the tool was to provide individualised practical information, signpost to reputable information sources, and enhance communication between patients and healthcare professionals.

Eighteen out of thirty in-patients consented to participant in the pilot evaluation with 15/18(83%) completing the study. Median (range) age was 66(22–85) years. The majority were male (10/15;66%). Pre-intervention, patients reported desiring further information regarding their infections and antibiotic therapy, including side effects of treatment. Deployment of the intervention improved short term knowledge and understanding of individuals infections and antibiotic management with median (IQR) scores improving from 3(2–5)/13 to 10(6–11)/13. 13/15(87%) reported that they would use the intervention again.

**Conclusion:**

A personalised, patient-centred intervention improved understanding and short-term knowledge of infections and antibiotic therapy in participating patients’. Long term impact on attitudes and behaviours post discharge will be further investigated.

**Electronic supplementary material:**

The online version of this article (10.1186/s13756-018-0333-1) contains supplementary material, which is available to authorized users.

## Background

Patient-centred interventions are important for ensuring appropriate, effective, safe, and responsive provision of healthcare [1]. When considering antibiotic prescribing and drug-resistant infections, the role of engaging patients and sharing in decision making around antibiotic prescribing can reduce the use of antibiotics in primary care [2]. In secondary care, there remains a paucity of data to guide development of patient-centred interventions that support patient engagement in the decision making process for antibiotic prescribing and infection management.

The management of acute infection in secondary care is often seen as a discrete episode. Recent evidence has demonstrated that poor communication and information provision during these episodes can have a cumulative effect on future attitudes and behaviours of patients towards infections and antibiotic use [3]. To investigate this, we worked with patients who had received antibiotics in hospital to co-design a patient-centred intervention. This intervention would aim to promote better communication and information provision during admission to hospital about an individual’s infection and antibiotic therapy.

This study was developed as part of a wider project aiming to develop and integrate several patient- and prescriber-focused interventions to support antibiotic decision making during infection management. This project is an integrated electronic clinical decision support tool entitled Enhanced, Personalised, Integrated Care for Infection Management at the Point-of-Care (EPIC IMPOC). This tool integrates the ability to interface with data from patient electronic health records, data visualisation, and a range of machine learning tools to support individualised antibiotic selection during infection management [4–6]. However, there is also a need to explore promoting more holistic approaches to evidence based antibiotic prescribing within decision support tools [7]. This includes the integration of patient-facing tools that can help to promote better understanding about an individual’s infection and its management [3, 7]. Through integration of these different modules, our aim is to improve patient engagement with the decision making process. Thus ensuring that patient views and preferences can be taken into account and that future attitudes and behaviours towards antimicrobials become more appropriate [3].

We report the development and pilot evaluation of a patient-centred intervention, co-designed with patients and embedded within EPIC IMPOC, to improve patient knowledge and understanding about the management of their infections and promote greater engagement with infection management.

## Method

### Patient workshops

**Fig. 1:**
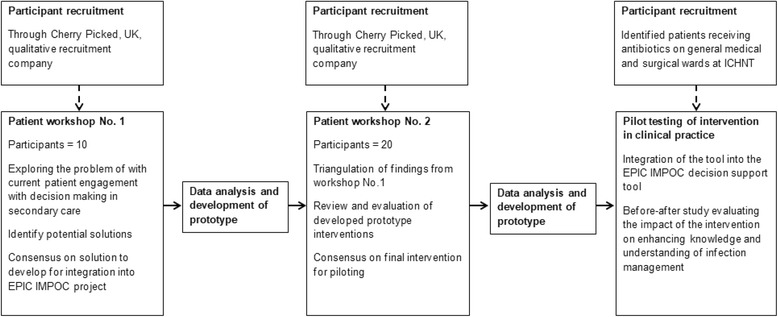
Summary of intervention development and pilot testing

Figure 1 summarises the process of intervention development employed within this study. In total, 30 previous patients who had received antibiotics in hospital during the preceding 12 months (recruited through *Cherry Picked*, UK – a specialist qualitative recruitment company) participated in two separate 1-h workshops. The first of these was held in September 2015 and the second in May 2016. Participants were recruited from a sample of 500 people whose data were held within a database of 20,000 individuals from around the UK who had signed up with the recruitment agency previously. An initial email was sent to all individuals in the database advertising the workshops. Respondents were then stratified according to recruitment criteria and 30 individuals selected for inclusion (10 were selected for the first workshop and 20 for the second). The primary participant recruitment criteria for inclusion was that the patient had received antibiotics in hospital within the preceding 12 months. We also aimed to select an equal spread of age ranges (18–24; 25–49; 50–65; 65+), gender, and ethnicities for the workshops. Following the initial invitation email, two further emails were sent to these individuals confirming their participation.

The first workshop, containing two focus groups, aimed to explore and co-design an outline of the intervention. This workshops outline criteria were based on the findings of a previously published study exploring current failures in communication and information provision around antibiotic prescribing and infection management in secondary care [3]. The second workshop contained a different group of participants and involved three focus groups. This aimed to triangulate the findings from the initial workshop and refine the design of the intervention that had been outlined by the participants at the first workshop. The number of groups per workshop (i.e. two and three groups, respectively) were selected to optimise the number of individuals within the focus groups and facilitate triangulation within workshops. Within workshops, the objectives of the different focus groups were identical.

Both workshops were facilitated by researchers trained in qualitative methodology (TMR, LSPM, EC) who used a standardised topic guide to explore and develop the intervention in focus groups of 5–7 participants (Additional files 1 & 2). Participants were consented and the workshops were audio-recorded. Anonymous recordings were transcribed verbatim for analysis. Independent researchers (ECS & BH) acted as observers for each focus group providing written observations to allow for greater reflexivity during data analysis.

Data were analysed using NVIVO Pro 11.0 software. For data analysis, a thematic framework was developed based on the findings of a previous study [3]. One researcher (TMR) initially reviewed all transcripts and data generated during the workshops. Line-by-line coding was then performed by two independent researchers (TMR & LSPM) with new emerging themes added to the framework in an inductive fashion [8]. During line-by-line coding the comments provided by the observers were reviewed as well as any written information produced by the groups during the workshop [9, 10]. This was considered to help balance areas of reflexivity derived from the coders’ own background, experiences, and beliefs [11]. After comparing coded transcripts at each interval of development, a list of categories were generated and members of the research team met to agree on key categories and themes that would inform the iterative design and refinement of the intervention. The intervention is described below in detail, but briefly, took the form of a personalised PDF document embedded within the electronic clinical decision support system, EPIC IMPOC. This allowed automated generation of PDF leaflets containing patient specific information on their antibiotic and infection management.

### Pilot study

Following workshops 1 and 2, the co-designed intervention was piloted in Imperial College Healthcare NHS Trust (ICHNT). This pilot aimed to provide an initial evaluation of the potential impact of the intervention and ascertain patient feedback on further improvements required before larger studies could be undertaken. The pilot involved a pre- and post-intervention questionnaire delivered 12–24 h either side of the intervention. This was developed based on the research groups previous experience of evaluating patient knowledge and understanding surrounding antimicrobial therapy and antimicrobial resistance [3, 12, 13]. The questionnaires were then piloted on two healthcare professionals, two members of the public, and a member of the research department not involved with this study.

Delivery of the intervention and questionnaires was facilitated by two members of the research team (TMR and VA). Participants were purposefully identified by clinical members of staff for inclusion from separate clinical wards across three university teaching hospitals making up ICHNT. These wards were staffed by a range of specialties (infectious diseases, care of the elderly, respiratory, gastroenterology, haematology, nephrology, general surgery, urology, and orthopaedics).

Over a four-week period, between 7th August and 1st September 2017, 30 in-patients were invited to participate in the intervention. Consent was obtained from patients who agreed to participate by members of the research team; they remained enrolled in the study for 3 days. After obtaining consent, participants were asked to complete a 15-point questionnaire on day one (Additional file 3). On day two a member of the research team, following a pre-determined script designed to simulate a discussion on infections / antibiotic prescribing during a ward round or brief clinical consultation (lasting less than 5 min), delivered the intervention. On day three, the participants were asked to complete a 20-point questionnaire (Additional file 3). The questionnaires were designed by the research team and were piloted on two healthcare professionals, four citizens not associated with the research team, and a medical student. The study was designed to assess (i) any short-term improvements in patient knowledge and understanding of their infection and antibiotics; (ii) what information was still being missed during the intervention; and (iii) evaluate the acceptability and agreement of patients with the intervention. Where answers were marked as correct/incorrect, members of the research team met and agreed upon correct responses for the individual participant before deployment of the questionnaire. Free text answers were collected and independently analysed inductively by two members of the research team (TMR and LSPM).

Data were anonymously collected and analysed using SPSS 24.0 and NVIVO pro 11.0 software. Local regional ethics committee approval was gained to undertake this study (*REC 17/LO/0047)*.

## Results

### Patient workshops

There was consistency in identified themes across both workshops. Participants agreed upon the development of a personalised PDF document that could be generated using electronically available data specific to the individual. Participants reported that the PDF was the optimal approach as it allowed the maximum flexibility to either be printed and given to a patient at the bedside or transferred electronically. Other approaches considered included the development of a mobile application, text message services, and written summaries. The ability to be able to print the PDF was considered by participants to address some of the reported concerns about transferring confidential patient information electronically and would also be available for patients without access to electronic devices.

*“Couldn’t you have an interactive PDF so people can choose whether or not to include a list of side effects or just the link for further information?”* (Female 1, workshop 1)

*“I like the idea of getting it electronically and downloading PDFs or something, but I would say an app’s just getting a little bit to gimmicky”* (Male 1, workshop 2)

*“I feel like that a lot of people prefer forms as they can physically keep track of them [patient information]. I feel more in control of them then. If you are comfortable online then it is good, however with medical records I mean they are quite sensitive, so it might be nice to have them just in their paper form.”* (Male 2, workshop 1)

Participants reported that the intervention could act as an important tool for promoting better communication about infections and antibiotic management between patients and healthcare professionals. In particular, participants reported that this may act as a prompt for further questions and support reflection on their infection and its management after the consultation has taken place.

*“I usually get home and think ‘oh wait’ I had a really important question which I forgot to ask. I like to be able to process things and then kind of gather my thoughts and find out what I want to know about the issue.”* (Male 3, workshop 1)

Table 1 summarises the key themes that emerged from the workshops for the content and structure the participants felt was required from the intervention. There was agreement across both workshops that the information provided needed to be personalised to the individual patient’s current situation and treatment regime. Existing approaches, such as medication information leaflets, were reported to give generic information on infections and treatments that participants felt could be overwhelming and confusing. Participants reported the need to ensure that the quantity and complexity of information provided was at a level that could be understood by the majority of individuals. To address this, the workshops decided that a summary of key points to take away should be presented with links to reputable information sources for patients to seek further information if required.

**Table 1:** Key themes identified during workshops for the development of a patient engagement intervention for promoting enhanced communication and information provision surrounding infection management in secondary care

Category	Summary of workshops decision on content	Summary of workshops decision on structure
Platform	Needed to be flexible, to allow use on devices, paper, in and out of hospital, and by all age groups The platform should also be personalisable, to allow the patient and doctor to select relevant information depending on the patient’s wishes	A PDF document that can be populated, printed, emailed, or uploaded onto an application was preferred. Mobile applications, websites, automated text systems were also considered but were felt not to have the same level of flexibility.
Individualised	The intervention should provide information about the individual’s current condition and treatment.	Information provided should be in summary form. The provision of blood test results, or probabilities was not felt to be appropriate as it could be overwhelming and concerning to some patients.
Health literate	The information must be provided in language that the majority of citizens can understand. The quantity of information provide must be enough to provide key information but not overwhelming to someone who is unwell and in hospital.	Colours and tables were not preferred. Participants opted for the minimum amount of presented information. Basic explanations of conditions with examples of medical terminology sometimes used was felt to be helpful for following discussions and searching for further information after the consultation.
Sign post	Detailed descriptions should not be included, but references for reputable sources of information should be provided to help guide those who want more information.	Links to further information on reputable websites. Blood test results were not preferred on the leaflet.
Practical advice	Advice on common or important side effects of treatments should be included. Practical information, such as whether it is okay to drink alcohol, drive/operate heavy machinery, and interactions with the oral contraceptive pill whilst taking antibiotics should be included. Educational information to promote better understanding of the risks of drug resistant infections could be included. Adherence to therapy should be reinforced.	Minimal numbers of side effects were preferred. The group decided on 3–4 key side effects would be optimal. A short description of antimicrobial resistance and where to find further information was included for reference.
A tool to enhance communication	The intervention should aim to enhance communication between patient and healthcare professionals. It should be designed to be delivered by all types of health care professional. It should provide a prompt to allow the patient to consider whether they have further questions, allowing them to pick this up during future interactions with the healthcare professional.	Diagnosis, causative organism, and treatments (past and present) were included.
Supporting follow up	Information on next appointments Information on who to contact if you have problems or questions on discharge	Removed from the leaflet as participants felt that it overlapped with discharge summaries that are often provided. In this case duplication of information at different times during hospital stay may be unhelpful.

*“Summarise it and then if you want more information you can always go on the internet”* (Female 2, workshop 2)

*“Rather than it being ‘here’s all the information in one go’, more of the ‘you have had a positive bacteria reading on your test, click here to read more about it’. And then if you don’t want to [don’t] click there”* (Female 3, workshop 2)

Furthermore, the groups focused on providing practical information that they felt was commonly missed during discussions with healthcare professionals regarding their medications in hospital. This included items such as whether it is safe to drink alcohol or drive whilst taking certain antibiotics.

“W*hen I had an infection and they said don’t take it if you have reflux – that stuff I need to know. But stuff like the massive long name of what the drug is really called and stuff like that…. It is irrelevant to me. I just want to know, is it going to make me sick? Can I drive? Can I work with it…”* (Male 4, workshop 1)

Participants reported that this this information needed to be provided in a health literate format that considered the literacy and language needs of the population who would be utilising this intervention.

*“Yes English as a second language and dyslexia is really common.”* (Female 4, workshop 1)

*“[It needs to be] easy to understand, easy to just [look down it]. Whereas here, I would look at this and say, well, this doesn’t, I don’t care because I can’t read this, I have no idea what this means”* (Female 5, workshop 2)

#### Intervention development

**Fig. 2:**
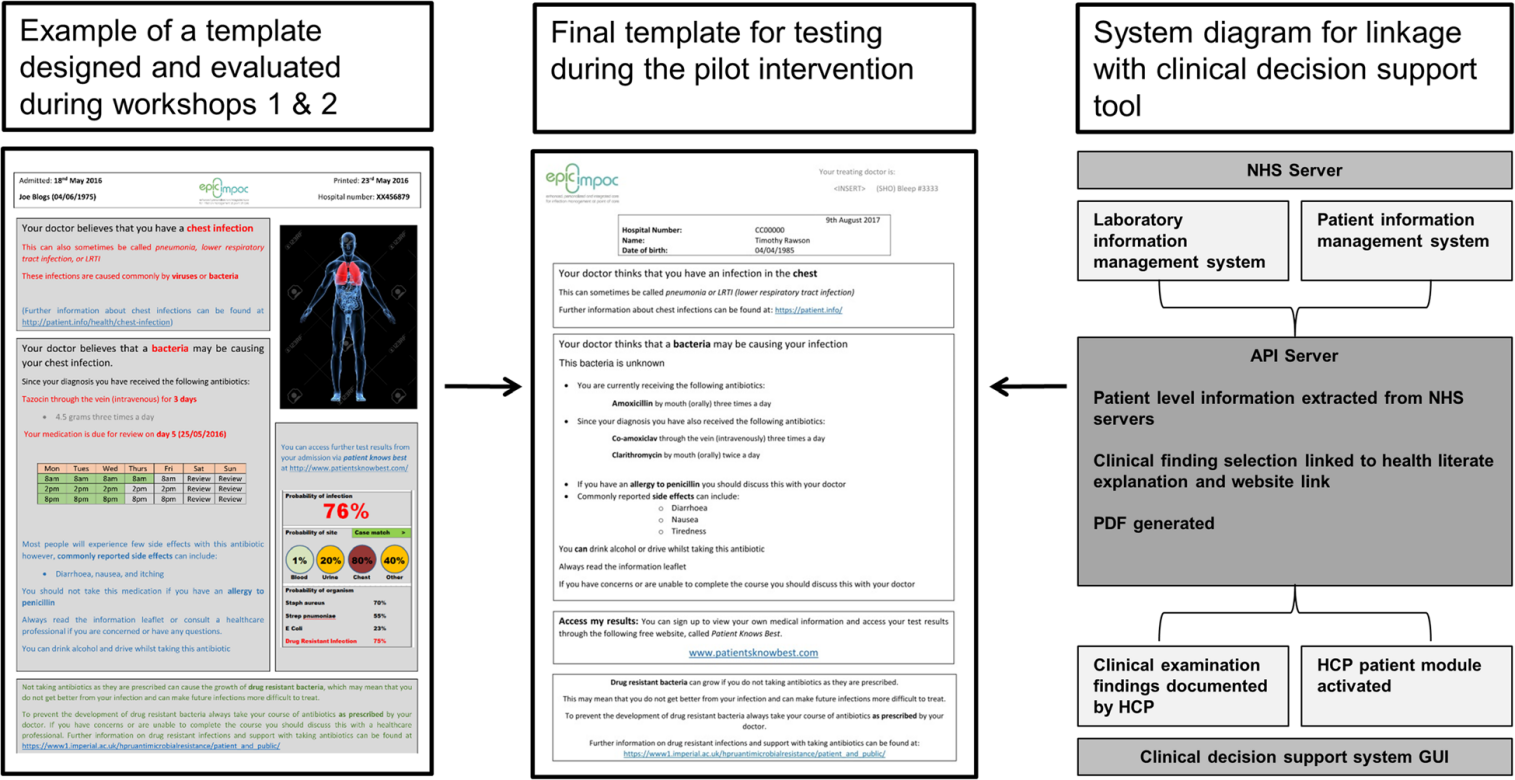
Summary of intervention development and linkage with clinical decision support system

Figure 2 demonstrates the final template that was agreed upon and co-designed by participants in the workshops. The intervention was embedded in an electronic clinical decision support system that contains several different modules linked to a central server. Although embedded with physician-facing decision support modules, this intervention currently sits independently of these. This allows individual patient information to be automatically extracted from a number of databases within the hospital. Moreover, the clinician can also input their impression and findings based on the clinical examination. To ensure that individualised information was provided in a health literate format, a number of translations automatically occur upon generation of the personalised information leaflet. For example, if “pneumonia” is recorded by the healthcare professional, it will be coded to display the diagnosis as “chest infection”. Moreover, a number of alternative names are provided (“pneumonia”, “lower respiratory tract infection”) automatically below on the PDF document. This code also triggers the inclusion of a web address that directly links to an open access patient information leaflet on pneumonia (patient.info). Therefore, on generating the information leaflet through the clinical decision support tool, the clinician is able to provide a personalised information leaflet to the patient, which contains details of their own infection and treatment.

#### Pilot study

Eighteen out of thirty (60%) patients invited consented to participate. The 12 who declined to take part did not provide reasons for this. In total, 15/18 (83%) of the enrolled participants completed the study. One patient moved hospital before they could complete the pre-intervention questionnaire, one participant was discharged before completion of the post-intervention questionnaire, and one patient experienced an episode of delirium after completion of the pre-intervention questionnaire leading to him being withdrawn from the study.

Table 2 summarises participant characteristics from the study. Of the 17 participants who completed our pre-intervention questionnaire, the median (range) age was 60 (22–85) years, the majority of participants were male (11/17; 65%). Most patients were under the care of medical specialties within the hospital (13/17; 76%). In the pre-intervention questionnaire, 8/17 (47%) reported the correct infection diagnosis and 6/17 (35%) correctly named what antibiotics they were receiving. Participants reported that health care professionals had spent less than 10 min discussing their infection with them in 9/17 (53%) cases. Three out of seventeen (18%) did not report healthcare professionals discussing their infection with them at all and 7/17 (41%) reported that healthcare professionals had spent longer the 10 min discussing their infections with them. Only 5/17 (29%) reported healthcare professionals discussing their antibiotic therapy with them during this admission.

**Table 2:** Summary of participant characteristics and questionnaire results from the pilot evaluation of the patient-focused intervention

Characteristic	Description	Result
Age^a^	Median (range) years	60 (22–85)
Gender^a^	Male (%)	11 (65)
Reported time spent discussing infection prior to intervention^a^		
*Not discussed*	*n* = (%)	3 (18)
*< 10 min*	*n* = (%)	8 (47)
*10–30 min*	*n* = (%)	3 (18)
*> 30 min*	*n* = (%)	3 (18)
Antibiotic therapy discussed with patient prior to intervention^a^		
*Yes pre-intervention*	*n* = (%)	5 (29)
Pre-intervention knowledge and understanding scores		
	Median (IQR)	3 (2–5)
	Mean (SD)	3.2 (2.2)
Post-intervention knowledge and understanding scores		
	Median (IQR)	10 (6–11)
	Mean (IQR)	8.5 (3.3)
Reported usefulness of intervention	Median score (range) 1 = Not very useful 6 = Extremely useful	5 (3–6)
Would participants use the intervention again	Yes – *n* = (%)	13 (87)
Reported optimal time to deploy the intervention		
*Initiation of therapy*	*n* = (%)	5 (33)
*On discharge*	*n* = (%)	2 (13)
*Any time during admission*	*n* = (%)	8 (53)

Legend: All analysis was performed only on participants with both pre- and post-questionnaires (*n* = 15) unless otherwise stated

^a^ *n* = 17 who completed pre-intervention questionnaire

Of the 15 patients that completed the study, the pre-intervention questionnaire demonstrated poor knowledge and understanding surrounding participant infections and antimicrobial therapy. Mean (SD) scores out of 13 were 3.2 (2.2). Following the intervention, participants post-intervention questionnaire scores improved to 8.5 (3.3) out of 13. Feedback on the impact of the questionnaire was positive with participants rating its usefulness a median (range) 5 (3–6) out of 6. Thirteen out of fifteen (87%) participants reported that they would use the intervention again if in hospital with an infection.

Table 3 summarises the questions participants recorded in their pre-intervention questionnaire regarding their infections and subsequent management. It also summarises participant post-intervention written feedback, outstanding questions, and suggestions for further development of the tool. Pre-intervention, participants reported requiring more information about their infections and antibiotic therapy than they had been given. Potential side effects were commonly reported questions that patients had. Post-intervention, participants reported that the intervention was useful as it provided information that had not yet been given to them by their treating doctor. This included information about their infection, the antibiotics that they were taking, and general issues around whether it is safe to drink alcohol or drive whilst taking these medications. Feedback provided on improvements to the intervention by participants surrounded, giving further information on specific aspects within the document and also prompting more detailed discussion with the doctor following use of this intervention.

**Table 3:** Summary of survey qualitative question responses from participants

Questions noted by participants pre-intervention	Frequency
What are the side effects of taking antibiotics?	7
Where to find further information about the diagnosis?	7
Further information about the antibiotics that I am taking	5
Further information about the bacteria causing my infection	3
How long will it take for me to feel better?	2
How can I prevent this happening again in the future?	2
Post-intervention - Why was this useful?	Frequency
It gave information I haven’t of been told by the doctor	4
I didn’t know the names of the antibiotics I was taking	3
Gave information about side effects	2
It provided information about driving	2
It provided information about drinking alcohol with antibiotics	2
Covered all of the questions that I wanted to ask the doctor	2
Gave information on the infection / bug	1
Clear and understandable information	1
A good reminder of my conversation with the doctor	1
Post-intervention - How could this be improved further?	Frequency
Nothing	3
More information on side effects	2
Would be better with more communication from the healthcare professional	1
Length of treatment	1
A place to write the concerns and questions that I have	1
Provide further information on why I shouldn’t drink on this medication	1

## Discussion

Within this study we have demonstrated that a patient-centred intervention, co-designed with patients to promote engagement with infection management in secondary care, improved participant knowledge and understanding of their infection in the short term. Participants responded positively to the intervention, providing data to triangulate findings from previous workshops, and providing feedback on future areas that still require development before wider deployment and evaluation.

Within secondary care there is evidence to demonstrate that both healthcare professionals and patients desire individuals to have a greater involvement with their medications during their in-patient stay [14]. This can help reduce medication errors and promote improved patient reported outcomes following hospital stay for a wide range of medications [14–16]. Given our groups previous observation of a desire from patients for better information about antibiotic therapy and the potential impact this may have on attitudes and behaviours, it is likely that such interventions could have a similar benefit as seen with other medications [3].

This study has highlighted the lack of awareness within our population regarding their infection and antibiotic therapy. Recall of infection names and antibiotic therapy were less than 50%. Less than 30% of patients remembered their healthcare professionals discussing their antibiotic therapy with them. There is a paucity of data to allow comparison of these findings with other similar studies of in-patients in secondary care. Micallef and colleagues, previously reported on the levels of awareness and understanding of antibiotic resistance and stewardship in a cohort of 1450 citizens attending hospital out-patient clinics and pharmacies in the UK [13]. Within this study, the authors identified broad conceptions about the development of drug-resistant infections and appropriate antibiotic use [13]. These findings have also been reported in community public awareness surveys that have demonstrated poor awareness and understanding surrounding antibiotics and infection management across a number of different countries [17]. We are now planning to undertake a further cross-sectional analysis of this problem to assess the levels of awareness of in-patients both with and without infections.

Within primary care, there is evidence supporting the role of shared decision making for reducing inappropriate antibiotic use [2, 18–20]. However, in secondary care during acute infection management, the need for antibiotic therapy is often a lot clearer, patients are more unwell, and decisions must be made rapidly, especially in the case of sepsis [3, 21, 22]. Therefore, when providing information on infections in secondary care interventions may need to adopt a different approach compared to primary care, where there often is truly a shared approach to making a final decision on the need for therapy. This problem has been addressed by Edwards and colleagues, who argue that engagement of the patient in decision making alone may be sufficient to improve understanding and involvement in the process overall, providing a level of ownership to the problem, whilst not requiring the focus to be on the final decision that is made [23]. This was supported by our findings that participants felt more informed and engaged with the management of their infections following the intervention, regardless of whether they had a final say in the decision that was made. Moreover, feedback on the intervention was overall very positive with the majority of participants happy to use the intervention again in future. However, a wide variation was observed with the preferred timing of the intervention reported by participants. This triangulates with findings from our development workshops, where there was variation in opinion between participants was observed on this topic.

The main finding from the workshops in this study was the reported focus on providing individualised information to patients that is relevant to their own specific situation. We were able to achieve this through the integration of this tool with a wider electronic clinical decision support tool, EPIC IMPOC. This allows us to utilise available electronic patient data and clinical examination findings recorded by the patient’s physician and provided a flexible mechanism of generating a personalised information leaflet for deployment at any point during the patient’s hospital stay. Despite this module not directly influencing physician-facing decision support modules within this study, there is a wider need to ensure that interventions are joined up during the development of clinical decision support tools, which are often developed with a narrow focus on antimicrobial selection only [7]. Validation of this intervention will now allow it to be tested in tandem with prescriber-focused interventions within the integrated decision support system.

A further aspect that participants in this study valued was ensuring that the intervention could be used by any healthcare professional, not just physicians. The role of healthcare professionals, such as nurses and pharmacists, is critical in infection management and appropriate antibiotic use [24–28]. Therefore, any tool that is developed must keep this in mind. Within our pilot study researchers of two different backgrounds successfully delivered the intervention. One was a nurse (VA) and the other a junior physician (TMR).

This study also had its limitations. As this was a pilot study that aimed to evaluate the intervention and provide feedback on further improvements before larger evaluation, only a small number of participants were recruited. To try to reduce bias of outcomes, patients were recruited from a wide number of wards and specialties, so that one clinical team did not heavily influence the outcome of this study. Secondly, the pilot only took place in three West London hospitals. Therefore, it may be difficult to generalise this study to wider populations. However, to address this the development workshops recruited from a large national database, with participants attending from many regions in south England. Thirdly, the questionnaire only aimed to assess short term improvement in knowledge and understanding. It is not possible to determine from this whether there would be any medium to long term impact from the intervention. Furthermore, the reported lengths of discussion with healthcare professionals about infections and antibiotics may not have been accurate given the subjectivity of participant reporting. However, this was felt to be appropriate within this study given that we were assessing the participant perceptions of information provision and communication with healthcare professionals. Finally, this pilot study was not powered to demonstrate statistical significant between pre- and post-intervention questionnaires. A larger, controlled study is now planned to assess the short, medium, and long-term impact of this intervention of participants receiving antibiotics in secondary care.

## Conclusion

Within our study we have observed poor baseline knowledge of antibiotic therapy and infection management amongst in-patients being treated for infections. Patients are accepting of simple, individualised information leaflets that can be delivered during routine clinical interactions. Such an intervention, co-designed by patients and embedded within a clinical decision support system was able to improve short term knowledge and understanding of antibiotic therapy and infection management within patients included in our study. This supports the need for greater emphasis on the development of patient-centred interventions to improve engagement with infections and their management in secondary care. Further work is required to quantify the short, medium, and long-term impacts of such interventions on patient knowledge, understanding, and attitudes towards antibiotic therapy.

## Additional files

Additional file 1: Topic guide used during workshop 1. (DOCX 52 kb)

Additional file 2: Topic guide used during workshop 2. (DOCX 23 kb)

Additional file 3: Questionnaire used before and after delivery of the pilot intervention. (DOCX 97 kb)
