# Supplementary material for: Development of a patient-centred intervention to improve knowledge and understanding of antibiotic therapy in secondary care
Source: Antimicrob Resist Infect Control. 2018 Mar 20;7:43. doi: 10.1186/s13756-018-0333-1 (PMC5859655; doi:10.1186/s13756-018-0333-1)
Supplement: Supplementary file 2 — Topic guide used during workshop 2. (DOCX 23 kb) [file 13756_2018_333_MOESM2_ESM.docx]

EPIC IMPOC session – TOPIC GUIDE

**Requirements:**

- Abstract from BMJ Open and figures
- Mock ups for discussion
- Current information leaflets
- Observer to take notes
- Split into two groups

**Introduction:**

*In September 2015 we held a workshop with 10 members of the public to explore their experience of being involved in decision making around management of infections they have been treated for in hospital. This showed us that clinicians and healthcare workers often fail to provide you with the information that you want to know and in a format that allows you to digest it when you are feeling unwell. This appeared to lead to misunderstandings about antibiotics and how to use them during future episodes as well.*

*The participants have worked with us to develop an idea about how we should deliver this information to help promote your engagement in the decision making process and today we would like you to help us develop this further over the next 30 minutes.*

| Time | Aim | Question plan |
| --- | --- | --- |
| *0-5min* | **Introduction** | Introduction and tell us whether you have ever received antibiotics from a hospital or GP (brief statement)  **Outline of paper findings and figures**   - **PDF format – flexible (can be printed / emailed / opened on mobile device)** - **Allows personalised information so not general like** - **NOT MEANT TO BE REPLACEMENT FOR PHARMA LEAFLET** |
| *5-10min* | **Confirm participant agreement with these findings** | - *Do you agree with the groups views from the previous workshop we held?* - *Would you suggest anything that is different we should focus on / improve?* |
| *10-25min* | **Hand out of mock ups for consideration**  **Work through hand outs**  **Explore whether this is helpful as a standalone information leaflet** | - *Please imagine that you are in hospital and have just sat down with the doctor. They have told you that you have a chest infection and are going to give you some antibiotics. You are given this information sheet by the doctor who tells you to have a read and let them know if you have any questions.* - *Explore:*   *Is the wording correct?*  *What information is missing? Is there too much information?*  *Are the links to other sources of information helpful?*  *Is the layout logical and easy to understand?*  *How would you change this?*   - *Would this help increase your understanding of your treatment?* - *When would you want to receive this? At the time you are given the antibiotic or on discharge?* - *Do you feel as if it would help you to question or challenge decisions about your management?* - *Should a healthcare worker go through this with you or would it be helpful if you were left to consider it by yourself?*   *If so who should / could go through it with you?*   - *Would this add to your understanding of what your infection and management?* |
| *25-30min* | **Confirm findings** | *Participants confirm the best format for presenting data*  *Participants confirm how this information sheet should be used to promote their engagement in the decision process*  *Participant agreement on what time this should be delivered* |
