# Supplementary material for: Development of a patient-centred intervention to improve knowledge and understanding of antibiotic therapy in secondary care
Source: Antimicrob Resist Infect Control. 2018 Mar 20;7:43. doi: 10.1186/s13756-018-0333-1 (PMC5859655; doi:10.1186/s13756-018-0333-1)
Supplement: Supplementary file 3 — Questionnaire used before and after delivery of the pilot intervention. (DOCX 97 kb) [file 13756_2018_333_MOESM3_ESM.docx]

**EPIC IMPOC – Patient engagement module – pre-intervention survey**

Thank you for agreeing to take part in the following survey. At Imperial College, researchers have been working with patient groups to develop a simple intervention to improve the way that healthcare professionals provide information to patients. Please complete the following questions without assistance from any member of staff. If you are unsure please leave the space blank or mark the answer with a cross. Thank you once again for your time and support.

1. What is the name if the infection you are being treated for?

**________________________________________________________________________**

1. What is the name of the organism causing the infection that you are being treated for (e.g. E.coli)

**________________________________________________________________________**

1. I am currently being given the following antibiotic(s) for my infection
   1. **Name(s)**

**__________________________________________________________________**

- 1. **Doses (amount)**

**__________________________________________________________________**

- 1. **Length (number of days)**

**__________________________________________________________________**

1. Side effects I have been warned about include

**________________________________________________________________________**

1. I could drink alcohol with this (these) antibiotic(s): **True / False / Unsure**
2. I could drive whilst taking this (these) antibiotic(s):  **True / False / Unsure**
3. What do you understand by the term “antimicrobial resistance” or “drug resistant infection”

**________________________________________________________________________________________________________________________________________________**

1. How long have the doctors/nurses/pharmacists caring for you spent talking to you about your infection and its treatment:

**they haven’t / <10 minutes / 10-30 minutes / >30 minutes**

1. Has the doctor provided all the information about your infection that you wanted to know?

**Yes / No / Unsure**

1. What outstanding questions do you have?

**______________________________________________________________________________________________________________________________________________________________________________________________________________________**

1. Has the doctor provided you with information about the medication (antibiotics) you are receiving?

**Yes / No / Unsure**

1. What outstanding questions do I have?

**______________________________________________________________________________________________________________________________________________________________________________________________________________________**

1. On discharge from the hospital will you have to continue taking antibiotics?

**Yes / No / Unsure**

1. If so, for how long?

**______________________________________________________________________**

1. When will you have to see a doctor about your infection after being discharged?

**______________________________________________________________________**

1. Will this be your GP or a doctor at the hospital?

**________________________________________________________________________**

**EPIC IMPOC – Patient engagement module – post-intervention survey**

Thank you for agreeing to take part in the following survey. At Imperial College, researchers have been working with patient groups to develop a simple intervention to improve the way that healthcare professionals provide information to patients. Please complete the following questions without assistance from any member of staff. If you are unsure please leave the space blank or mark the answer with a cross. Thank you once again for your time and support.

1. What is the name if the infection you are being treated for?

**________________________________________________________________________**

1. What is the name of the organism causing the infection that you are being treated for (e.g. E.coli)

**________________________________________________________________________**

1. I am currently being given the following antibiotic(s) for my infection
   1. **Name(s)**

**__________________________________________________________________**

- 1. **Doses (amount)**

**__________________________________________________________________**

- 1. **Length (number of days)**

**__________________________________________________________________**

1. Side effects I have been warned about include

**________________________________________________________________________**

1. I could drink alcohol with this (these) antibiotic(s): **True / False / Unsure**
2. I could drive whilst taking this (these) antibiotic(s):  **True / False / Unsure**
3. What do you understand by the term “antimicrobial resistance” or “drug resistant infection”

**________________________________________________________________________________________________________________________________________________**

1. How long have the doctors/nurses/pharmacists caring for you spent talking to you about your infection and its treatment:

**they haven’t / <10 minutes / 10-30 minutes / >30 minutes**

1. Has the doctor provided all the information about your infection that you wanted to know?

**Yes / No / Unsure**

1. What outstanding questions do you have?

**______________________________________________________________________________________________________________________________________________________________________________________________________________________**

1. Has the doctor provided you with information about the medication (antibiotics) you are receiving?

**Yes / No / Unsure**

1. What outstanding questions do I have?

**______________________________________________________________________________________________________________________________________________________________________________________________________________________**

1. On discharge from the hospital will you have to continue taking antibiotics?

**Yes / No / Unsure**

1. If so, for how long?

**______________________________________________________________________**

1. When will you have to see a doctor about your infection after being discharged?

**______________________________________________________________________**

1. Will this be your GP or a doctor at the hospital?

**_______________________________________________________________________**

1. Did you find the information leaflet useful?

**(Not at all) 1 2 3 4 5 6 (extremely)**

1. Why?

**______________________________________________________________________________________________________________________________________________**

1. What can be improved?

**______________________________________________________________________________________________________________________________________________**

1. Would you use this leaflet again?

**______________________________________________________________________________________________________________________________________________________________________________________________________________________**

1. When would the best time to be given this be?

**______________________________________________________________________________________________________________________________________________**
